# Structural *vs.* functional specialization: distinct competitive profiles in youth male and female flatwater paddlers

**DOI:** 10.7717/peerj.21388

**Published:** 2026-06-12

**Authors:** Weidong Gao, Yuanye Zhu, Weibing Ye, Jiale Chen, Qian Chen

**Affiliations:** 1 Zhejiang Institute of Sports Science, Hangzhou, Zhejiang Province, China; 2 School of Dance and Martial Arts, Capital University of Physical Education and Sports, Beijing, China; 3 College of Physical Education and Health Sciences, Zhejiang Normal University, Jinhua, Zhejiang Province, China

**Keywords:** Competitive characteristic profiles, Anthropometry, Youth athletes, Gender, Physiological adaptation, Core stability

## Abstract

**Objective:**

This study aimed to determine the extent to which structural (anthropometric) versus functional (physiological) characteristics drive the specialization of youth kayakers and canoeists.

**Methods:**

Using a cross-sectional comparative design, eighty-two youth athletes (42 kayakers, 40 canoeists) from the Zhejiang Provincial Training Camp were assessed. Comprehensive profiling included anthropometry (height, arm span), body composition, hematological markers (Hb, HCT), and specific fitness tests (Wingate anaerobic test, 1-RM strength, core stability, and 3,000 m run).

**Results:**

Distinct specialization patterns emerged, differentiated significantly by gender. Male athletes exhibited both structural and functional divergence: kayakers possessed significantly greater height, arm span, hematological capacity (Hb, HCT), and explosive peak power, whereas canoeists demonstrated superior posterior core stability, with no significant differences in anaerobic endurance between disciplines. In contrast, female athletes showed no significant anthropometric differences; divergence was exclusively functional. Foundational aerobic capacity and upper-body strength did not differ between female disciplines; instead, their specialization was highly specific to the glycolytic system, with female canoeists demonstrating superior anaerobic average power.

**Conclusion:**

The findings confirm that while male specialization is constrained by structural mismatches and distinct explosive power requirements, female specialization allows for greater talent mobility due to the lack of structural barriers, relying instead on highly specific functional adaptation within the anaerobic energy pathways.

## Introduction

Flatwater sprint kayaking and canoeing are Olympic disciplines that demand a complex combination of explosive power, high-level endurance, and refined technical skill. Athletes must produce and sustain tremendous force to overcome water resistance, requiring highly developed aerobic and anaerobic energy systems (Crisafulli et al., 2025). Success at the elite level is the culmination of years of structured training, and it begins with the early identification and development of young athletes who possess the requisite physical and physiological attributes for the sport (Baker et al., 2017). Understanding the key anthropometric and physiological characteristics that define a successful paddler is therefore fundamental to effective coaching and talent development programs, particularly during the critical adolescent growth period (Till, Scantlebury & Jones, 2017). According to the Long-Term Athlete Development (LTAD) framework, this adolescent phase (approx. 14–15 years of age) corresponds precisely to the critical transition from the ‘Training to Train’ to the ‘Training to Compete’ stages (Balyi, Way & Higgs, 2013). During this window, the LTAD model emphasizes a vital shift from generalized physical preparation toward highly discipline-specific specialization. Therefore, understanding the structural and functional divergence at this exact stage is crucial.

While often trained together and categorized within the same “paddling” group, kayaking and canoeing are distinct disciplines with fundamental biomechanical differences. Kayaking involves a seated position with a double-bladed paddle, promoting a symmetrical, rotational movement of the torso. In contrast, canoeing requires a high-kneeling stance, utilizing a single-bladed paddle on one side of the boat, which places a greater demand on core stability and asymmetrical power application. This functional asymmetry in canoeing is distinct from the symmetrical demands of kayaking, leading to different patterns of trunk muscle activation and strength requirements (Kendal & Sanders, 1992). These profound differences in technique and posture logically suggest that each discipline may cultivate, or select for, a unique set of anthropometric, physiological, and fitness characteristics.

Previous research has provided valuable profiles of paddlers, often focusing on the determinants of elite performance (Álvarez-Yates & García-García, 2021) or specific physiological aspects. For instance, extensive investigations have detailed the relationship between performance, body composition, and physical strength in elite sprint paddlers (Hamano et al., 2015), as well as specific trunk muscle responses (Kocjan & Šarabon, 2020). Similarly, parallel research has established performance prediction models for elite kayakers (Van Someren & Howatson, 2008) and strategies to optimize the demanding physiological training for canoeists (García-Pallarés & Izquierdo, 2011).

However, a significant limitation of this literature is its traditional focus on senior, elite-level athletes. While valuable research has begun to shift towards junior populations—such as analyzing the specific physiological traits of junior kayakers (Borges et al., 2015) or profiling young female sprint kayakers (Lopez-Plaza et al., 2019)—this work is often discipline-specific. Even studies that do directly compare young kayakers and canoeists often focus primarily on anthropometry (Alacid et al., 2015; Manna & Adhikari, 2018) or a combination of anthropometry and physical fitness (Gäbler et al., 2023; López-Plaza et al., 2017a; López-Plaza et al., 2017b). Despite this progress, a comprehensive, multi-dimensional analysis that simultaneously integrates detailed anthropometry with a full battery of sport-specific physical fitness tests (*e.g.*, upper/lower body strength, power, and specific endurance) is not yet fully established. Crucially, how these structural (anthropometric) and functional (physiological) traits interact with sex during the adolescent specialization process remains largely unexplored.

This gap highlights the critical necessity of a comprehensive comparative study. A deeper understanding of the differences and similarities at this key developmental age is essential for creating effective talent identification and development models (Baker et al., 2017). Furthermore, rather than examining longitudinal talent transitions, it is crucial to determine the extent to which structural prerequisites (low trainability) *versus* functional adaptations (high trainability) drive the divergence of athlete profiles during specialization.

Therefore, the primary objective of this cross-sectional study was to conduct a comprehensive comparative analysis of the anthropometric characteristics and sport-specific physical fitness of youth flatwater kayakers and canoeists. By systematically comparing a wide array of indices, we aim to delineate the distinct “competitive characteristic profiles” for each discipline. Based on the fundamental biomechanical differences between the two disciplines, we hypothesized that the specialization profiles would be dual-layered, distinguishing between inherent structural prerequisites (anthropometry) and highly trainable functional adaptations (physical fitness). Furthermore, we hypothesized that these structural and functional specialization patterns would be significantly modulated by sex. The findings are intended to provide an evidence-based framework to inform talent identification and guide specialization pathways.

## Materials & Methods

### Participants

A total of 82 adolescent athletes were recruited to participate in this study. All participants were attendees of the 2024 Zhejiang Provincial Canoeing and Kayaking Training Camp. The Zhejiang provincial team represents a premier elite training center in China, having historically produced multiple Olympic and World champions in flatwater sprint disciplines. Therefore, while drawn from a single province, this cohort reflects a highly representative sample of elite Chinese youth paddlers. The sample size represented the total available cohort meeting inclusion criteria at the training camp. To justify this sample size, a sensitivity power analysis was conducted using G*Power (version 3.1.9.7). For a two-way analysis of variance (ANOVA) with an *α* level of 0.05 and statistical power (1-*β*) of 0.80, the available sample of *N* = 82 provides sufficient statistical power to reliably detect a medium-to-large interaction effect (*f* = 0.315, equivalent to *η*_p_^2^ ≈ 0.09). The cohort included 42 kayakers (30 male, 12 female) and 40 canoeists (18 male, 22 female, Table 1). Inclusion criteria required a minimum of three years of systematic training experience in their respective discipline. All participants were in good health and had been free from any major injuries for at least six months prior to the study.

**Table 1:** Demographic summary of participants by sport discipline.

	**Kayakers**	**Canoeists**	**Total**	*p*-value
**Number of participants (n)**				
Male	30	18	48	
Female	12	22	34	
Total	42	40	82	
**Age (years), mean ± SD (95% CI)**				
Male	15.07 ± 1.08 (14.67–15.47)	14.78 ± 1.26 (14.15–15.41)	14.96 ± 1.15 (14.63–15.29)	0.404
Female	14.50 ± 1.24 (13.71–15.29)	14.50 ± 1.01 (14.05–14.95)	14.50 ± 1.08 (14.12–14.88)	0.999
Total	14.90 ± 1.14 (14.54–15.26)	14.63 ± 1.16 (14.26–15.00)	14.77 ± 1.14 (14.52–15.02)	0.268

**Notes.**

Values for age are presented as mean ± standard deviation (95% confidence interval). The *p*-value indicates the statistical difference in age between the kayaker and canoeist groups.

The study protocol was approved by the Institutional Review Board of Zhejiang Normal University (Approval No. ZSRT2024276). Written informed consent was obtained from all participants and their legal guardians before the commencement of any testing procedures.

### Study design and procedure

A cross-sectional, comparative study design was employed. The entire testing protocol was conducted over three consecutive days. Day 1 was for accommodation and briefing. Testing occurred on Day 2. To facilitate the testing flow, participants were divided into smaller subgroups to rotate through stations following an identical, standardized schedule, sequenced to minimize fatigue contamination (Haff & Triplett, 2021). The testing schedule was officially announced to all coaches and athletes one month in advance to ensure psychological and physical preparation. Furthermore, a formal reminder was issued to the coaching staff three days prior to the testing to facilitate appropriate training load adjustments.

### Morning (06:30–07:30)

Participants reported to the laboratory after a minimum 10-hour overnight fast for a baseline venous blood draw. This ensures that metabolic and hormonal markers (*e.g.*, cortisol, testosterone) are in a stable, basal state, free from the confounding effects of recent food intake or strenuous exercise (Lee et al., 2017). Additionally, athletes were required to refrain from strenuous physical training for 24 h preceding the blood collection.

### Post-breakfast (09:00–11:30)

Following a standardized breakfast and an adequate rest period (approx. 90–120 min), the first testing battery began. This session included tests highly sensitive to fatigue and was ordered as follows.

Anthropometry and body composition: Non-fatiguing tests such as height, arm span, and bioelectrical impedance analysis (BIA) were conducted first.

Core stability tests: The battery of core stability tests was performed next. These muscular endurance tests must be completed while the athlete is fresh (pre-fatigue) to represent a true maximum capacity (Vera-Garcia, Grenier & McGill, 2000). A five-minute rest period was administered between each directional test.

### Afternoon (14:00–16:30)

After a standardized lunch and a 2–3 h rest and digestion period, the maximal-effort tests were conducted. The sequence followed established guidelines, moving from maximal power/strength (neural) to maximal endurance (metabolic) (Haff & Triplett, 2021; Sporis et al., 2009).

### Measurements

#### Anthropometry and body composition

Standing height, sitting height, arm span, and various circumferences were measured using a stadiometer and a non-elastic tape measure strictly following the standardized protocols outlined in the Chinese national guidelines for sports measurement and evaluation (Sun, Sun & Chen, 2022). To eliminate inter-rater variability, all physical measurements were consistently performed by the same experienced researcher. Body mass and composition variables (*e.g.*, body fat percentage, skeletal muscle mass) were assessed using a bioelectrical impedance analysis device (Inbody 720; Biospace, Seoul, South Korea). Participants were instructed to stand barefoot on the device, wearing light clothing, and follow standardized procedures to ensure measurement accuracy.

#### Physiological markers

Fasting blood samples were collected *via* venipuncture into EDTA and serum-separating tubes. Hematological parameters, including red blood cell count (RBC), hemoglobin (Hb), and hematocrit (HCT), were analyzed using a Sysmex XT-2000i automated hematology analyzer (Sysmex Corporation, Kobe, Japan). Serum biochemical markers, including urea, testosterone, and creatine kinase (CK), were analyzed with a Beckman AU480 automated chemistry analyzer (Beckman Coulter, Brea, CA, USA). Serum cortisol was measured using a Beckman ACCESS 2 immunoassay system.

#### Anaerobic power

A 30-second maximal anaerobic power test (Wingate test) was performed on a Wattbike Pro cycle ergometer (Nottingham, UK). Participants completed a standardized 5-minute low-intensity warm-up, interspersed with two 5-second maximal sprints for neuromuscular activation. Following a 3-minute passive recovery, the 30-second maximal test was initiated against a constant resistance of 7.5% body mass. Key metrics, including peak power (W), average power (W), and a fatigue index, were recorded for analysis. Relative power values (W/kg) were calculated by dividing power outputs by body mass.

#### Strength and endurance

Maximal upper-body strength was determined *via* one-repetition maximum (1-RM) tests for the bench press and seated cable row (bench pull), strictly adhering to the National Strength and Conditioning Association (NSCA) youth resistance training guidelines. To ensure safety and data integrity, all 1-RM protocols were conducted under one-on-one supervision by certified coaches, with participation restricted to athletes possessing at least three years of systematic training experience. Upper-body muscular endurance was evaluated using a one-minute maximum pull-up test, while aerobic capacity was assessed *via* a 3,000-meter run on a standard 400-meter track. Finally, core stability was quantified through a battery of timed isometric holds—including the prone bridge, supine bridge, and bilateral side bridges—following the protocols validated by McGill, Childs & Liebenson (1999). These tests are recognized for their high reliability in assessing torso muscular endurance in youth athletic populations. The duration in seconds was recorded for each position, with tests terminated immediately upon the athlete’s failure to maintain the standardized posture despite verbal cues.

### Statistical analysis

All data were processed and analyzed using JASP (Version 0.19.0, JASP Team, 2024). Descriptive statistics (mean ±standard deviation & 95% confidence interval) were calculated to summarize participant characteristics and performance metrics. Prior to inferential testing, all variables were assessed for normality and homogeneity of variance using the Shapiro-Wilk and Levene’s tests, respectively. To properly assess the main effects of sex and discipline, as well as their interaction (Sex × Discipline), a two-way ANOVA was utilized. Effect sizes for the ANOVA were reported as partial eta-squared (*η*_p_^2^). For variables that severely violated the assumption of homogeneity of variances (*e.g.*, Average Power, Bench Press), a log-transformation was applied prior to the ANOVA to meet statistical assumptions. Crucially, our analytical approach was adapted based on the specific data distributions and interaction outcomes. When a significant Sex × Discipline interaction effect was observed (*e.g.*, Wingate parameters), simple main effects analyses with Bonferroni corrections were conducted to isolate the specific disciplinary differences within each sex cohort. Furthermore, for specific variables where severe heteroscedasticity persisted or could not be adequately resolved by transformation (*e.g.*, Supine Bridge, Bench Pull), the non-parametric Mann–Whitney *U* test was strictly employed to robustly verify independent differences between disciplines within the same sex. The threshold for statistical significance was established at *p* < 0.05.

## Results

Baseline demographic analysis (Table 1) confirmed no significant age differences across the sex and discipline subgroups (*p* > 0.05).

### Anthropometric and body composition characteristics

A two-way ANOVA was utilized to assess differences in anthropometric and body composition profiles, with a Log10 transformation applied to body mass to address heteroscedasticity (Levene’s *p* improved from 0.010 to 0.145).

**Figure 1:**
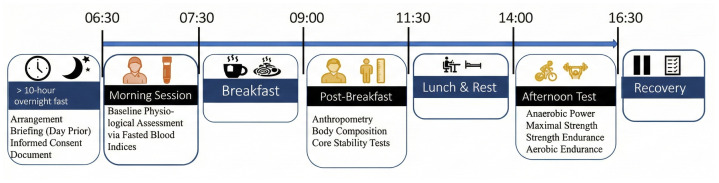
Schematic representation of the experimental protocol.

As detailed in Table 2, a significant main effect of sex was observed across all measured structural and compositional variables, including body mass index (BMI) and body fat percentage (all *p* < 0.05), reflecting expected sexual dimorphism. Furthermore, significant main effects of discipline were identified for arm span, shoulder width, and waist circumference (all *p* < 0.05), indicating that kayakers generally exhibited broader upper-body dimensions than canoeists. These representative structural differences are visually depicted in Fig. 1. Crucially, no significant Sex × Discipline interactions were found for any anthropometric or body composition variables (all *p* > 0.05). As illustrated by the parallel estimated marginal means lines in Fig. 1, these consistent overarching trends suggest that fundamental structural and compositional dimensions act as universal biological prerequisites, devoid of sex-specific divergence between the two paddling disciplines.

**Table 2:** Two-way ANOVA of anthropometric and body composition parameters across sex and discipline.

Variables	Male Kayakers	Male Canoeists	Female Kayakers	Female Canoeists	Sex *p* (*η*_p_^2^)	Discipline *p* (*η*_p_^2^)	Interaction *p* (*η*_p_^2^)
Height (cm)	180.2 ± 4.1	177.4 ± 5.2	167.6 ± 4.7	167.1 ± 3.6	**<0.001** (0.62)	0.102 (0.03)	0.287 (0.02)
Body Mass^*^ (kg)	74.7 ± 8.1	72.1 ± 7.1	60.85 ± 5.6	59.85 ± 4.2	**<0.001** (0.50)	0.266 (0.02)	0.672 (<0.01)
Sitting Height (cm)	96.4 ± 3.1	96.1 ± 3.0	89.7 ± 2.4	90.2 ± 2.1	**<0.001** (0.55)	0.814 (<0.01)	0.531 (<0.01)
Arm Span (cm)	184.5 ± 4.8	180.2 ± 4.7	169.3 ± 5.6	168.4 ± 5.6	**<0.001** (0.62)	**0.033 (0.06)**	0.159 (0.03)
Shoulder Width (cm)	41.4 ± 1.6	40.6 ± 1.6	37.6 ± 1.3	36.7 ± 1.3	**<0.001** (0.60)	**0.017 (0.07)**	0.972 (<0.01)
Waist Circ. (cm)	79.0 ± 5.2	77.1 ± 4.5	72.1 ± 4.3	69.5 ± 3.0	**<0.001** (0.39)	**0.032 (0.06)**	0.748 (<0.01)
Hip Circ. (cm)	95.4 ± 4.4	93.6 ± 4.6	92.1 ± 3.3	91.9 ± 4.1	**0.013** (0.08)	0.310 (0.01)	0.396 (0.01)
BMI (kg/m^2^)	23.0 ± 2.3	23.0 ± 2.0	21.7 ± 1.9	21.5 ± 1.7	**0.004** (0.10)	0.883 (<0.01)	0.763 (<0.01)
Body Fat %	10.4 ± 3.1	11.6 ± 4.9	18.5 ± 3.8	19.3 ± 3.7	<**0.001** (0.50)	0.308 (0.01)	0.844 (<0.01)

**Notes.**

Data are presented as mean ± SD. Bold values indicate statistical significance at *p* < 0.05. *η*_p_^2^ = partial eta-squared.

* The variable was analyzed using a Log10 transformation to meet the assumption of homoscedasticity, although original descriptive data are presented here for clarity and clinical relevance.

### Biochemical markers

As detailed in Table 3, all hematological, metabolic, and endocrine markers exhibited significant main effects of sex (*p* < 0.05), reflecting standard biological dimorphism. Notably, significant main effects of discipline were observed within the hematological profile (RBC, hemoglobin, hematocrit) and basal testosterone levels, with kayakers consistently demonstrating higher baseline values than canoeists (all *p* < 0.05). Conversely, stress and metabolic load indicators, including blood urea and cortisol, showed no significant discipline effects (*p* > 0.05). Furthermore, no significant Sex × Discipline interactions were detected across any physiological variables. These findings objectively indicate that while baseline metabolic stress remains comparable between the two disciplines, kayaking is associated with a higher baseline requirement for oxygen-carrying capacity and its associated anabolic endocrine profile, independent of sex.

**Table 3:** Two-way ANOVA of biochemical parameters across sex and discipline.

Variables	Male Kayakers	Male Canoeists	Female Kayakers	Female Canoeists	Sex *p* (*η*_p_^2^)	Discipline *p* (*η*_p_^2^)	Interaction *p* (*η*_p_^2^)
RBC (×10^12^/L)	5.16 ± 0.31	5.03 ± 0.30	4.70 ± 0.31	4.54 ± 0.28	**<0.001** (0.38)	**0.037** (0.05)	0.865 (<0.01)
Hemoglobin (g/dL)	14.99 ± 0.62	14.51 ± 0.98	13.74 ± 0.79	12.93 ± 0.67	**<0.001** (0.46)	**<0.001** (0.15)	0.335 (0.01)
Hematocrit (%)	46.38 ± 1.93	44.28 ± 2.39	41.89 ± 1.59	41.21 ± 1.81	**<0.001** (0.47)	**0.003** (0.11)	0.125 (0.03)
Blood Urea (mmol/L)	6.08 ± 1.09	6.26 ± 1.03	5.18 ± 0.92	5.38 ± 0.94	**<0.001** (0.15)	0.419 (<0.01)	0.968 (<0.01)
Cortisol (ng/mL)	17.36 ± 4.45	15.21 ± 2.74	17.84 ± 2.43	19.36 ± 5.51	**0.022** (0.07)	0.751 (<0.01)	0.067 (0.04)
Testosterone (ng/dL)^*^	549.2 ± 135.6	486.2 ± 99.0	50.1 ± 15.0	36.7 ± 13.5	**<0.001** (0.94)	**0.003** (0.11)	0.134 (0.03)

**Notes.**

Data are presented as mean ± SD. Bold values indicate statistical significance at *p* < 0.05.

*η*_p_^2^ = partial eta-squared.

* The variable was analyzed using a Log10 transformation to meet the assumption of homoscedasticity, original descriptive data are presented here for clarity.

**Table 4:** Two-way ANOVA of anaerobic power, aerobic endurance, strength, and core stability.

Variables	Male Kayakers	Male Canoeists	Female Kayakers	Female Canoeists	Sex *p* (*η*_p_^2^)	Discipline *p* (*η*_p_^2^)	Interaction *p* (*η*_p_^2^)
Peak power (W)	918.4 ± 144.9^a^	799.2 ± 102.7	585.3 ± 64.2	624.8 ± 82.5	**<0.001** (0.55)	0.131 (0.03)	**0.003** (0.11)
Average power (W)^*^	575.7 ± 70.2	552.5 ± 61.0	339.7 ± 25.7^b^	373.2 ± 46.2	**<0.001** (0.79)	0.364 (0.01)	**0.020** (0.07)
RPP (W/kg)	12.21 ± 1.36^a^	11.03 ± 1.18	9.63 ± 1.11	10.36 ± 1.19	**<0.001** (0.29)	0.449 (0.01)	**0.001** (0.12)
RAP (W/kg)	7.68 ± 0.69	7.62 ± 0.58	5.58 ± 0.62^b^	6.20 ± 0.56	**<0.001** (0.65)	0.058 (0.05)	**0.024** (0.06)
3,000 m Run Time (s)	698.7 ± 55.8	645.3 ± 169.8	756.6 ± 59.4	800.2 ± 77.9	**<0.001** (0.21)	0.839 (<0.01)	**0.048** (0.05)
1-RM Bench Press (kg)^**^	95.83 ± 22.59	93.61 ± 25.08	55.91 ± 9.95	55.56 ± 15.52	**<0.001** (0.48)	0.595 (<0.01)	0.990 (<0.01)
1-RM Bench Pull (kg)^**^	93.33 ± 17.44	84.17 ± 18.17	59.09 ± 5.39	54.22 ± 11.78	**<0.001** (0.51)	0.063 (0.05)	0.564 (<0.01)
1-min Pull-ups (reps)	38.23 ± 11.06	39.00 ± 14.74	33.09 ± 12.62	24.79 ± 10.77	**0.002** (0.13)	0.223 (0.02)	0.144 (0.03)
Prone Bridge (s)	201.9 ± 45.7	212.0 ± 57.8	290.1 ± 80.4	280.9 ± 77.7	**<0.001** (0.27)	0.976 (<0.01)	0.530 (0.01)
Supine Bridge (s)^**^	87.0 ± 22.4^a^	161.3 ± 143.6	150.7 ± 38.2	167.8 ± 87.3	0.090 (0.04)	**0.029** (0.06)	0.166 (0.03)
Left Side Bridge (s)	131.6 ± 40.7	131.4 ± 60.0	177.4 ± 47.5	184.4 ± 48.7	**<0.001** (0.20)	0.774 (<0.01)	0.763 (<0.01)
Right Side Bridge (s)	152.5 ± 60.3	174.3 ± 79.3	208.6 ± 62.3	196.4 ± 56.5	**0.015** (0.08)	0.759 (<0.01)	0.283 (0.02)

**Notes.**

Data are presented as Mean ± SD.

RPP Relative Peak Power RAP Relative Average Power

Bold values indicate statistical significance at *p* <  0.05. *η*_p_^2^ = partial eta-squared.

a A significant difference compared to Male Canoeists within the same sex (simple main effect, *p* < 0.05).

b A significant difference compared to Female Canoeists within the same sex (simple main effect, *p* < 0.05).

* Analyzed using Log10-transformed data due to heteroscedasticity.

** Due to severe heteroscedasticity, ANOVA models for Bench Press were based on log-transformed values. Furthermore, the absence of discipline effects for Bench Pull, and the specific discipline differences in the Supine Bridge, were robustly confirmed *via* non-parametric Mann–Whitney *U* tests.

### Anaerobic power performance in the wingate test

**Figure 2:**
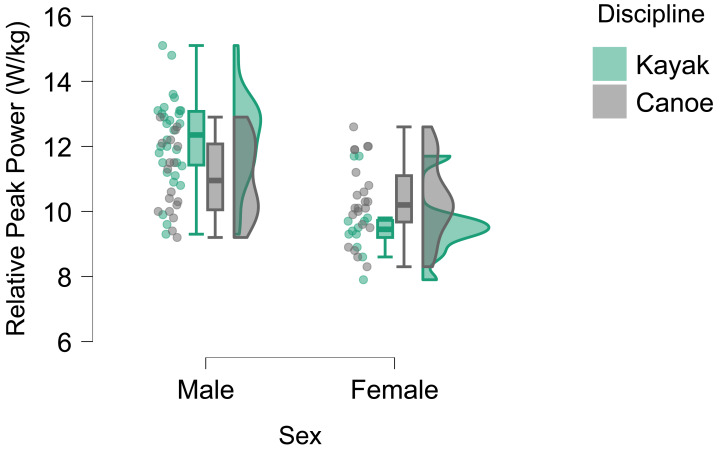
Sex and discipline differences in relative peak power.

**Figure 3:**
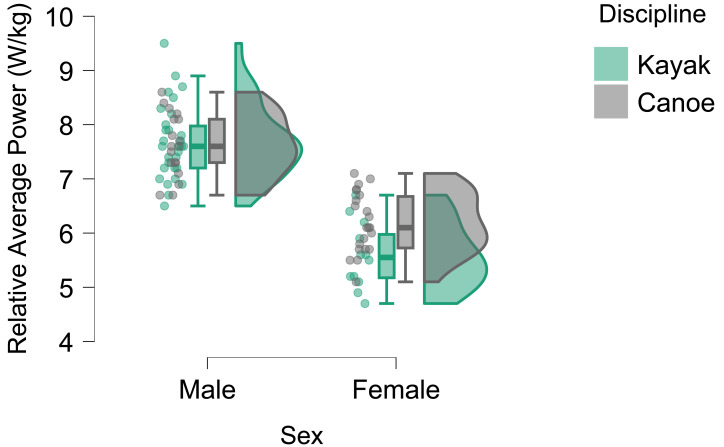
Sex and discipline differences in relative average power.

Table 4 summarizes the descriptive statistics and two-way ANOVA results for all physiological tests. For the Wingate anaerobic test, significant main effects of sex were observed across all parameters (all *p* < 0.001), with males outperforming females. Crucially, significant sex-by-discipline interactions were identified for peak power (PP; *p* < 0.05), relative PP (*p* < 0.05), log-transformed average power (AP; *p* < 0.05), and relative AP (*p* < 0.05). Simple main effects analysis revealed a double dissociation pattern: for peak power indices (phosphagen system), male kayakers scored significantly higher than male canoeists (*p* < 0.01, Fig. 2), while female athletes showed no disciplinary differences. Conversely, for average power indices (glycolytic system), female canoeists significantly outperformed female kayakers (*p* < 0.05, Fig. 3), whereas male athletes exhibited no significant differences between disciplines. This double dissociation pattern is visually confirmed by the individual data distributions in the raincloud plots (Figs. 2 and 3).

### Aerobic endurance performance

Regarding aerobic endurance, a significant main effect of sex was observed for the 3,000 m run time (*p* < 0.001, *η*_p_^2^ = 0.21), with male athletes completing the test significantly faster than female athletes. The main effect of discipline was not significant (*p* = 0.839). Although a marginal sex-by-discipline interaction was detected (*p* < 0.05), subsequent simple main effects analyses revealed no statistically significant disciplinary differences within either the male (*p* = 0.076) or female (*p* = 0.255) cohorts, indicating that general aerobic endurance is determined primarily by sex rather than the specific canoe sprint discipline.

### Strength and core stability

Upper-body absolute strength and muscular endurance were assessed *via* the 1RM bench press, bench pull, and pull-up tests. Highly significant main effects of sex were observed across all three strength metrics (all *p* < 0.05, Table 4), favoring male athletes. However, no significant main effects of discipline or sex-by-discipline interactions were identified, which was further confirmed *via* non-parametric tests due to heteroscedasticity in the bench pull data.

Core stability was evaluated using prone, supine, and bilateral side bridge tests. For the prone and bilateral side bridges, significant main effects of sex were consistently observed (*p* < 0.05), with female athletes demonstrating significantly longer hold times than males across these metrics, independent of discipline. Conversely, for the supine bridge (posterior chain), non-parametric Mann-Whitney *U* tests revealed a divergent disciplinary pattern: male canoeists demonstrated substantially greater hold times than male kayakers (*p* < 0.05), whereas no significant difference was observed between female kayakers and canoeists (*p* = 0.964).

## Discussion

This study conducted a comprehensive comparative analysis of youth flatwater kayakers and canoeists, revealing that distinct, highly specialized competitive profiles emerge even at the adolescent stage. Our findings provide a nuanced perspective on athlete development, indicating that the nature of this divergence differs significantly by gender. For male athletes, this specialization manifests as a profound difference in both anthropometric and functional characteristics. For female athletes, the divergence was almost exclusively functional, centered on a highly specific double dissociation within their anaerobic energy systems.

### Anthropometric constraints and biomechanical stability

The most prominent finding in male athletes was the anthropometric divergence: kayakers were significantly taller and possessed a greater arm span. This result is not isolated; it confirms findings from other international cohorts, which also identify male kayakers as possessing greater size and reach (Alacid et al., 2015; Hagner-Derengowska et al., 2014). From a biomechanical perspective, this divergence likely stems from the fundamental stability differences between the two disciplines. The kayak, characterized by a seated position, a lower center of gravity, and the inherent stabilizing effect of a double-bladed paddle, provides a comparatively stable platform (Kocjan & Šarabon, 2020). This stability allows taller athletes with greater arm spans to effectively leverage their reach to maximize stroke length and power generation (Van Someren & Howatson, 2003).

Conversely, the canoe is defined by its inherent instability, requiring rapid trunk muscle reflex responses to manage the high center of gravity and constant rotational forces generated by the high-kneeling, asymmetrical stroke (Kocjan & Šarabon, 2020). This “instability-first” demand may actively select against the very tall frames seen in kayakers, as a more compact build is advantageous for balance. This hypothesis also provides a compelling explanation for our gender-specific findings. In males, who are on average taller with longer limbs, the disparity in center-of-gravity height between a ‘seated’ *vs.* ‘high-kneeling’ position is far more extreme, making stability a critical limiting factor. In female athletes, this postural center-of-gravity shift is less pronounced, potentially reducing the selection pressure on height and allowing functional characteristics to become the primary drivers of specialization.

The fundamental mechanisms driving these distinct structural constraints and developmental trajectories between sexes can be largely attributed to sex-specific endocrine profiles during puberty (Senefeld & Hunter, 2024). In males, the surge in endogenous androgens, particularly testosterone, powerfully stimulates muscle protein synthesis and bone mineralization, leading to a disproportionate increase in upper-body muscle mass, broader biacromial breadth, and superior maximal strength outputs (Joyner, Hunter & Senefeld, 2025). Conversely, the estrogenic environment in females promotes earlier epiphyseal growth plate closure, which typically results in a comparatively shorter final stature and limits the absolute divergence in structural metrics. Furthermore, increased estrogen levels facilitate distinct female adiposity patterns and induce anatomical adaptations, such as increased pelvic width, which inherently affect lever lengths and force production efficiency (Kodete et al., 2024). However, it is important to note that hormonal profiles were not directly quantified in the present study. Therefore, these mechanistic explanations remain theoretical within our specific cohort, underscoring the need for future longitudinal investigations that integrate direct endocrine assessments to substantiate these growth patterns in elite youth paddlers.

### Physiological adaptations: the “engine” models

Functionally, our data reveals distinct, discipline-specific “engine” models. For male athletes, the divergence is rooted in the phosphagen system and posterior chain stability. The male canoeist’s profile is a direct adaptation to their unstable environment. To transfer power during the asymmetrical, unilateral C-1 stroke, the athlete’s trunk must first become a rigid platform. Our finding of superior posterior chain core endurance (supine bridge) in male canoeists is the physical manifestation of this “stability-first” model.

In contrast, the male kayaker model is built upon explosive instantaneous power. Our Wingate test results demonstrated that male kayakers possess significantly higher absolute and relative peak power (PP), identifying the phosphagen system as a key differentiator. Critically, this high-power output is built upon a superior aerobic “chassis”. The significantly higher levels of hemoglobin (Hb) and hematocrit (HCT) found in kayakers suggests a greater oxygen-carrying capacity, essential for recovery and direct energy contribution in sprint kayaking (Zouhal et al., 2012). Furthermore, it is noteworthy that a marginally significant interaction trend (*p* = 0.067) suggested a tendency for male kayakers to exhibit higher baseline cortisol levels compared to male canoeists. Cortisol is often used as a biomarker to assess training load and physiological stress. In this context, this tendency may reflect the distinct training architectures of the two disciplines: kayaking typically demands a higher volume of systemic aerobic conditioning (a known driver of elevated baseline cortisol), whereas canoe training is more heavily weighted towards explosive anaerobic power. While this difference might reflect such distinct chronic training stress, it could also be attributed to acute psychological anticipation of the upcoming physical tests or variations in sleep quality prior to data collection. However, as this study employed a cross-sectional design, it cannot determine whether this is a result of long-term training adaptation or the influence of short-term factors prior to testing. Therefore, the underlying mechanism for this finding requires further clarification through longitudinal research.

### Functional specialization in female athletes

Among female athletes, the narrative shifts entirely. The absence of significant anthropometric differences is, in itself, a crucial finding, reinforcing our hypothesis that structural selection pressure is lower. Notably, foundational upper-body strength (bench press, bench pull, pull-ups) and general aerobic endurance (3,000 m run) did not significantly differ between female kayakers and canoeists. Instead, the divergence is characterized by a highly specific adaptation in the glycolytic system.

Female canoeists demonstrated significantly superior absolute and relative average power (AP) during the Wingate test compared to female kayakers, while peak power remained similar. This indicates a clear specialization for anaerobic power-endurance. The kneeling position and single-bladed stroke of the canoe are biomechanically less efficient than the seated, double-bladed kayak stroke, thus requiring female canoeists to rely heavily on sustained glycolytic energy pathways to maintain high-torque, repetitive propulsion over the race distance (Álvarez-Yates & García-García, 2021). Additionally, it is worth noting that female athletes, regardless of discipline, exhibited significantly superior anterior and lateral core endurance (prone and side bridges) compared to their male counterparts, highlighting a gender-specific strategy for maintaining trunk stiffness during waterward force application.

### Practical implications and limitations

Placed within the context of the LTAD framework, these findings provide theoretical grounding for discipline orientation and physical conditioning during the ’Training to Compete’ stage (Balyi, Way & Higgs, 2013). For male athletes, discipline orientation should be structurally determined early in the LTAD pathway, as success in either kayak or canoe is hindered by fundamental anthropometric mismatches and distinct explosive power *versus* posterior-chain stability requirements. For female athletes, the lack of a structural barrier suggests that discipline orientation relies heavily on re-engineering the athlete’s dominant anaerobic energy system (shifting from a balanced profile to a highly glycolytic-dominant capacity).

This study has limitations. First, the cross-sectional design identifies existing differences but cannot establish causality—does specific training history shape the athlete, or do talent selection systems recruit athletes with these innate predispositions? A longitudinal study is required to clarify this (Baker et al., 2017). Second, due to the logistical constraints of conducting extensive testing within a provincial training camp setting, we were unable to assess biological maturation timing (*e.g.*, Tanner stages) or strictly control for the menstrual cycle phase in female athletes. Consequently, chronological age was utilized to define the youth cohort. Future studies should incorporate maturation tracking and hormonal profiling to isolate the precise effects of puberty on structural differentiation. Third, the sample was drawn from a single provincial training camp, and the findings may not be generalizable to all adolescent paddlers. Finally, the lack of direct biomechanical analysis (*e.g.*, *via* load cells, IMUs, or strain gauges on paddles) prevents a definitive link between these isolated physiological profiles and on-water stroke kinematics. Integrating such technologies remains a crucial direction for future research (Romagnoli et al., 2024).

## Conclusions

This comparative study demonstrates that youth flatwater kayakers and canoeists possess distinct and highly specialized competitive profiles, even at the adolescent stage of development. For male athletes, this divergence is characterized by a combination of anthropometric advantages and superior explosive peak power in kayakers (greater height and arm span) and superior posterior-chain core stability in canoeists, establishing two different athlete models. Among female athletes, the profiles diverge primarily along functional lines, but rather than differing in general aerobic or strength capacities, their specialization is highly specific to the glycolytic system, with canoeists specialized for anaerobic power endurance.

## Supplemental Information

10.7717/peerj.21388/supp-1 Supplemental Information 1 Raw dataset of anthropometric, physiological, and physical fitness characteristics for all 82 participants

10.7717/peerj.21388/supp-2 Supplemental Information 2 STROBE statement checklist

## Abbreviations

The following abbreviations are used in this manuscript:

BIA: Bioelectrical Impedance Analysis
BMI: Body Mass Index
CK: Creatine Kinase
Hb: Hemoglobin
HCT: Hematocrit
LTAD: Long-Term Athlete Development
RAP: Relative Average Power
RBC: Red Blood Cell Count
RPP: Relative Peak Power
1-RM: One-Repetition Maximum

## Additional Information and Declarations

### Data Availability

The following information was supplied regarding data availability: The raw data is available in the Supplemental Files.

## References

[ref-1] Alacid F, Marfell-Jones M, Muyor JM, Martínez I (2015). Kinanthropometric comparison between young elite kayakers and canoeists. Collegium Antropologicum.

[ref-2] Álvarez-Yates T, García-García O (2021). Determinants of flatwater canoeing and kayaking performance: a systematic review. Medicina Dello Sport.

[ref-3] Baker J, Cobley S, Schorer J, Wattie N (2017). Talent identification and development in sport: an introduction. Routledge handbook of talent identification and development in sport.

[ref-4] Balyi I, Way R, Higgs C (2013). Long-term athlete development.

[ref-5] Borges TO, Dascombe B, Bullock N, Coutts AJ (2015). Physiological characteristics of well-trained junior sprint kayak athletes. International Journal of Sports Physiology and Performance.

[ref-6] Crisafulli O, Fortunati M, Gemelli T, Febbi M, Drid P, Ramat S, D’Antona G (2025). Power–load relationship of bench press, ballistic bench press, and prone bench pull in international medal-winning canoeists and kayakers. Sports.

[ref-7] Gäbler M, Prieske O, Elferink-Gemser MT, Hortobágyi T, Warnke T, Granacher U (2023). Measures of physical fitness improve prediction of kayak and canoe sprint performance in young kayakers and canoeists. Journal of Strength and Conditioning Research.

[ref-8] García-Pallarés J, Izquierdo M (2011). Strategies to optimize concurrent training of strength and aerobic fitness for rowing and canoeing. Sports Medicine.

[ref-9] Haff G, Triplett N (2021). Essentials of strength training and conditioning.

[ref-10] Hagner-Derengowska M, Hagner W, Zubrzycki IZ, Krakowiak H, Słomko W, Dzierzanowski M, Rakowski A, Wiącek-Zubrzycka M (2014). Body structure and composition of canoeists and kayakers: analysis of junior and teenage Polish national canoeing team. Biology of Sport.

[ref-11] Hamano S, Ochi E, Tsuchiya Y, Muramatsu E, Suzukawa K, Igawa S (2015). Relationship between performance test and body composition/physical strength characteristic in sprint canoe and kayak paddlers. Open Access Journal of Sports Medicine.

[ref-12] Joyner MJ, Hunter SK, Senefeld JW (2025). Evidence on sex differences in sports performance. Journal of Applied Physiology.

[ref-13] Kendal SJ, Sanders RH (1992). The technique of elite flatwater kayak paddlers using the wing paddle. Journal of Applied Biomechanics.

[ref-14] Kocjan A, Šarabon N (2020). Increased liveliness of trunk muscle responses in elite kayakers and canoeists. Sports.

[ref-15] Kodete CS, Thuraka B, Pasupuleti V, Malisetty S (2024). Hormonal influences on skeletal muscle function in women across life stages: a systematic review. Muscles.

[ref-16] Lee EC, Fragala MS, Kavouras SA, Queen RM, Pryor JL, Casa DJ (2017). Biomarkers in sports and exercise: tracking health, performance, and recovery in athletes. Journal of Strength and Conditioning Research.

[ref-17] López-Plaza D, Alacid F, Muyor JM, López-Miñarro PÁ (2017a). Differences in anthropometry, biological age and physical fitness between young elite kayakers and canoeists. Journal of Human Kinetics.

[ref-18] López-Plaza D, Alacid F, Muyor JM, López-Miñarro PÁ (2017b). Sprint kayaking and canoeing performance prediction based on the relationship between maturity status, anthropometry and physical fitness in young elite paddlers. Journal of Sports Sciences.

[ref-19] Lopez-Plaza D, Alacid F, Rubio-Arias JA, Lopez-Minarro PA, Muyor JM, Manonelles P (2019). Morphological and physical fitness profile of young female sprint kayakers. Journal of Strength and Conditioning Research.

[ref-20] Manna T, Adhikari S (2018). A comparative study of anthropometric and physical profiles of male junior rowers, kayakers and canoers. Medicina Sportiva: Journal of Romanian Sports Medicine Society.

[ref-21] McGill SM, Childs A, Liebenson C (1999). Endurance times for low back stabilization exercises: clinical targets for testing and training from a normal database. Archives of Physical Medicine and Rehabilitation.

[ref-22] Romagnoli C, Boatto P, Campoli F, Caprioli L, Delgado D, Edriss S, Frontuto C, Lanotte N, Annino G, Padua E, Bonaiuto V (2024). Monitoring of kinetic parameters in sprint canoeing performance.

[ref-23] Senefeld JW, Hunter SK (2024). Hormonal basis of biological sex differences in human athletic performance. Endocrinology.

[ref-24] Sporis G, Jukic I, Ostojic SM, Milanovic D (2009). Fitness profiling in soccer: physical and physiologic characteristics of elite players. Journal of Strength and Conditioning Research.

[ref-25] Sun QZ, Sun JH, Chen PY (2022). Sports measurement and evaluation.

[ref-26] Till K, Scantlebury S, Jones B (2017). Anthropometric and physical qualities of elite male youth rugby league players. Sports Medicine.

[ref-27] Van Someren KA, Howatson G (2003). Prediction of 200-m sprint kayaking performance. Canadian Journal of Applied Physiology.

[ref-28] Van Someren KA, Howatson G (2008). Prediction of flatwater kayaking performance. International Journal of Sports Physiology and Performance.

[ref-29] Vera-Garcia FJ, Grenier SG, McGill SM (2000). Abdominal muscle response during curl-ups on both stable and labile surfaces. Physical Therapy & Rehabilitation Journal.

[ref-30] Zouhal H, Lahaye SLD, Abderrahaman AB, Minter G, Herbez R, Castagna C (2012). Energy system contribution to Olympic distances in flat water kayaking (500 and 1,000 m) in highly trained subjects. Journal of Strength and Conditioning Research.

