# Supplementary material for: Structural vs. functional specialization: distinct competitive profiles in youth male and female flatwater paddlers
Source: PeerJ. 2026 Jun 12;14:e21388. doi: 10.7717/peerj.21388 (PMC13267790; doi:10.7717/peerj.21388)
Supplement: Supplemental Information 2 [file peerj-14-21388-s002.pdf]

STROBE Statement—checklist of items that should be included in reports of observational studies

	Item No.	Recommendation	Page No.	Relevant text from manuscript
**Title and abstract**	1	(*a*) Indicate the study's design with a commonly used term in the title or the abstract	4	Using a cross-sectional comparative design
		(*b*) Provide in the abstract an informative and balanced summary of what was done and what was found	4	Objective: This study aimed ... Methods: ... included anthropometry... Results: Distinct specialization patterns emerged... Conclusion: The findings confirm...
**Introduction**				
Background/rationale	2	Explain the scientific background and rationale for the investigation being reported	5	Understanding the key anthropometric and physiological characteristics... is therefore fundamental... kayaking and canoeing are distinct disciplines with fundamental biomechanical differences
Objectives	3	State specific objectives, including any prespecified hypotheses	5	Therefore, the primary objective of this study was to conduct a comprehensive comparative analysis of the anthropometric characteristics and sport-specific physical fitness…
**Methods**				
Study design	4	Present key elements of study design early in the paper	7	A cross-sectional, comparative study design was employed.
Setting	5	Describe the setting, locations, and relevant dates, including periods of recruitment, exposure, follow-up, and data collection	7	All participants were attendees of the 2024 Zhejiang Provincial Canoeing and Kayaking Training Camp. ... The entire testing protocol was conducted over three consecutive days
Participants	6	(*a*) *Cross-sectional study*—Give the eligibility criteria, and the sources and methods of selection of participants	7	Inclusion criteria required a minimum of three years of systematic training

				experience... good health... free from any major injuries...
Variables	7	Clearly define all outcomes, exposures, predictors, potential confounders, and effect modifiers. Give diagnostic criteria, if applicable	8-9	Measurements: Anthropometry and Body Composition... Physiological Markers... Anaerobic Power... Strength and Endurance...
Data sources/ measurement	8*	For each variable of interest, give sources of data and details of methods of assessment (measurement). Describe comparability of assessment methods if there is more than one group	*8-9*	Inbody 720... Sysmex XT-2000i... Wattbike Pro... Stadiometer..." (Specific devices listed in Measurements section).
Bias	9	Describe any efforts to address potential sources of bias	8	..participants were divided into smaller subgroups... sequenced to minimize fatigue contamination... minimum 10-hour overnight fast...
Study size	10	Explain how the study size was arrived at	7	The sample size represented the total available cohort meeting inclusion criteria at the training camp.

Continued on next page

Quantitative variables	11	Explain how quantitative variables were handled in the analyses. If applicable, describe which groupings were chosen and why	9	Relative power values (W/kg) were calculated... Inter-group differences... were then analyzed…
Statistical methods	12	(*a*) Describe all statistical methods, including those used to control for confounding	10	All data were processed and analyzed using JASP... Kolmogorov-Smirnov... independent samples t-test... Mann-Whitney U test...
**Results**				
Participants	13*	(a) Report numbers of individuals at each stage of study—eg numbers potentially eligible, examined for eligibility, confirmed eligible, included in the study, completing follow-up, and analysed	10-11	The study included a total of 82 participants, with 42 kayakers and 40 canoeists... (Table 1)
Descriptive data	14*	(a) Give characteristics of study participants (eg demographic, clinical, social) and information on exposures and potential confounders	11	As shown in Table 1, there were no significant differences in age...
Outcome data	15*	*Cross-sectional study—Report numbers of outcome events or summary measures*	*11-12*	Reported in Tables 2, 3, 4, 5, (e.g., "male kayakers were significantly taller... and had a significantly greater arm span...")
Main results	16	(*a*) Give unadjusted estimates and, if applicable, confounder-adjusted estimates and their precision (eg, 95% confidence interval). Make clear which confounders were adjusted for and why they were included	11-12	Results are reported as Mean ± SD (95% CI) with p-values. e.g., "male kayakers had significantly higher levels of hemoglobin (Hb, $p = 0.045$) ...

Continued on next page

Other analyses	17	Report other analyses done—eg analyses of subgroups and interactions, and sensitivity analyses	N/A	-
**Discussion**				
Key results	18	Summarise key results with reference to study objectives	12-13	This study... revealing that distinct, highly specialized competitive profiles emerge even at the adolescent stage... divergence differs significantly by gender
Limitations	19	Discuss limitations of the study, taking into account sources of potential bias or imprecision. Discuss both direction and magnitude of any potential bias	15	However, this study has limitations. The cross-sectional design... sample was drawn from a single provincial training camp…
Interpretation	20	Give a cautious overall interpretation of results considering objectives, limitations, multiplicity of analyses, results from similar studies, and other relevant evidence	15	Anthropometric Constraints and Biomechanical Stability... Physiological Adaptations... The findings are intended to provide an evidence-based framework…
Generalisability	21	Discuss the generalisability (external validity) of the study results	15	...findings may not be generalizable to all adolescent paddlers.
**Other information**				
Funding	22	Give the source of funding and the role of the funders for the present study and, if applicable, for the original study on which the present article is based	17	Funding acquisition, Q.C.

*Give information separately for cases and controls in case-control studies and, if applicable, for exposed and unexposed groups in cohort and cross-sectional studies.

Note: An Explanation and Elaboration article discusses each checklist item and gives methodological background and published examples of transparent reporting. The STROBE checklist is best used in conjunction with this article (freely available on the Web sites of PLoS Medicine at http://www.plosmedicine.org/, Annals of Internal Medicine at http://www.annals.org/, and Epidemiology at http://www.epidem.com/). Information on the STROBE Initiative is available at www.strobe-statement.org.
